# Supplementary material for: The Bayesian confidence intervals for measuring the difference between dispersions of rainfall in Thailand
Source: PeerJ. 2020 Aug 6;8:e9662. doi: 10.7717/peerj.9662 (PMC7415225; doi:10.7717/peerj.9662)
Supplement: Supplemental Information 2 [file peerj-08-9662-s002.docx]

**Dataset S1**. Monthly rainfall data (mm.) measuring from Jana, Songkhla, Thailand from 2008 to 2017

|  | 2008 | 2009 | 2010 | 2011 | 2012 | 2013 | 2014 | 2015 | 2016 | 2017 |
| --- | --- | --- | --- | --- | --- | --- | --- | --- | --- | --- |
| January | 0.5 | 25.6 | 24.6 | 156.7 | 290.2 | 62.9 | 12.0 | 8.0 | 92.5 | 352.4 |
| February | 11.5 | 0.0 | 0.0 | 0.0 | 10.0 | 152.0 | 0.0 | 0.0 | 28.0 | 5.5 |
| March | 15.2 | 89.0 | 12.5 | 136.3 | 49.2 | 0.0 | 0.0 | 0.0 | 0.0 | 51.9 |
| April | 0.0 | 43.2 | 0.0 | 40.5 | 181.1 | 14.7 | 5.5 | 20.0 | 0.0 | 254.5 |
| May | 3.5 | 96.6 | 6.0 | 15.0 | 32.0 | 129.5 | 52.9 | 55.0 | 39.0 | 136.8 |
| June | 85.3 | 15.5 | 19.0 | 87.9 | 54.0 | 80.1 | 81.4 | 27.0 | 30.0 | 52.9 |
| July | 47.1 | 85.1 | 38.7 | 106.5 | 7.0 | 95.4 | 25.0 | 29.1 | 24.6 | 62.0 |
| August | 62.0 | 9.5 | 0.0 | 151.7 | 78.5 | 103.1 | 199.2 | 95.0 | 70.9 | 205.0 |
| September | 27.0 | 18.0 | 84.0 | 79.4 | 147.6 | 50.5 | 60.0 | 145.5 | 70.8 | 153.3 |
| October | 49.9 | 30.7 | 297.3 | 101.6 | 154.6 | 162.7 | 443.8 | 185.0 | 127.1 | 165.1 |
| November | 713.9 | 678.7 | 388.0 | 444.1 | 104.7 | 581.3 | 429.5 | 247.0 | 211.0 | 1017.3 |
| December | 267.8 | 41.4 | 372.7 | 640.5 | 272.0 | 324.1 | 573.0 | 49.2 | 656.7 | 269.9 |

**Note:** Data-sets of rainfall measuring from Jana, Songkhla, Thailand from 2008 to 2017 obtained from website of Southern Meteorological Center (East Coast).

(http://www.songkhla.tmd.go.th/rain/ampore/sjana.html)
