# Supplementary material for: The Bayesian confidence intervals for measuring the difference between dispersions of rainfall in Thailand
Source: PeerJ. 2020 Aug 6;8:e9662. doi: 10.7717/peerj.9662 (PMC7415225; doi:10.7717/peerj.9662)
Supplement: Supplemental Information 3 [file peerj-08-9662-s003.docx]

**Dataset S2**. Monthly rainfall data (mm.) measuring from Ranod, Songkhla, Thailand from 2008 to 2017

|  | 2008 | 2009 | 2010 | 2011 | 2012 | 2013 | 2014 | 2015 | 2016 | 2017 |
| --- | --- | --- | --- | --- | --- | --- | --- | --- | --- | --- |
| January | 830.6 | 0.0 | 0.0 | 1.6 | 56.8 | 428.3 | 85.3 | 0.0 | 32.2 | 177.2 |
| February | 26.3 | 40.7 | 0.0 | 0.0 | 152.5 | 0.0 | 0.0 | 0.0 | 5.0 | 37.0 |
| March | 20.7 | 0.0 | 0.0 | 0.0 | 0.0 | 37.5 | 193.5 | 0.0 | 92.0 | 0.0 |
| April | 29.4 | 0.0 | 38.2 | 0.0 | 107.4 | 5.5 | 26.7 | 0.0 | 81.0 | 66.7 |
| May | 23.1 | 21.5 | 41.5 | 62.2 | 157.5 | 5.5 | 0.0 | 0.0 | 47.5 | 60.5 |
| June | 0.0 | 16.2 | 0.0 | 0.0 | 77.3 | 26.4 | 0.0 | 0.0 | 0.0 | 28.1 |
| July | 0.0 | 7.2 | 25.0 | 0.0 | 18.8 | 0.0 | 6.7 | 8.0 | 8.5 | 15.6 |
| August | 1.2 | 5.0 | 12.5 | 36.0 | 62.7 | 32.0 | 6.0 | 0.0 | 6.8 | 79.8 |
| September | 105.0 | 0.0 | 76.1 | 18.4 | 8.7 | 39.9 | 16.7 | 8.0 | 0.0 | 5.4 |
| October | 54.0 | 44.0 | 279.7 | 197.5 | 251.9 | 216.5 | 311.1 | 314.8 | 42.0 | 89.0 |
| November | 1059.1 | 186.4 | 652.8 | 478.8 | 630.9 | 216.8 | 518.9 | 470.5 | 295.0 | 856.6 |
| December | 345.6 | 1324.5 | 125.0 | 473.5 | 291.1 | 279.1 | 406.9 | 381.2 | 25.1 | 223.3 |

**Note:** Data-sets of rainfall measuring from Ranod, Songkhla, Thailand from 2008 to 2017 obtained from website of Southern Meteorological Center (East Coast).

(http://www.songkhla.tmd.go.th/rain/ampore/sranod.html)
